# Mid-Trimester Fetal Anterior Abdominal Wall Subcutaneous Tissue Thickness: An Early Ultrasonographic Predictor of Gestational Diabetes Mellitus

**DOI:** 10.7759/cureus.34610

**Published:** 2023-02-03

**Authors:** Isha Seth, Ram K Aiyappan, Sunayana Singh, Aditya Seth, Deepti Sharma, Janu M K, Vivek Krishnan, Arushi Seth, Chander Mohan Yadav, Harsh Jain

**Affiliations:** 1 Obstetrics and Gynaecology, Amrita Hospital, Faridabad, IND; 2 General Surgery, Amrita Hospital, Faridabad, IND; 3 Obstetrics and Gynaecology, Pandit Bhagwat Dayal Sharma Post Graduate Institute of Medical Sciences, Rohtak, IND; 4 Orthopaedics, Pandit Bhagwat Dayal Sharma Post Graduate Institute of Medical Sciences, Rohtak, IND; 5 Obstetrics and Gynaecology, Amrita Institute of Medical Sciences, Kochi, IND; 6 Perinatology and Foetal Medicine, Amrita Institute of Medical Sciences, Kochi, IND; 7 Medicine, Jawaharlal Nehru Medical College, Belgaum, IND; 8 Orthopaedics and Rehabilitation, Pandit Bhagwat Dayal Sharma Post Graduate Institute of Medical Sciences, Rohtak, IND

**Keywords:** fetal anterior abdominal wall subcutaneous tissue thickness, mid-trimester scan, fasting blood sugar, gestational diabetes mellitus, fastt

## Abstract

Background

This study aimed to determine whether mid-trimester fetal anterior abdominal wall subcutaneous tissue thickness (FASTT) is an early sonographic predictor of gestational diabetes mellitus (GDM), as well as to study its correlation with maternal glycemic values on GDM screening at 24-28 weeks.

Methodology

We conducted a prospective, case-control study. FASTT was assessed at anomaly scan in 896 uncomplicated singleton pregnancies. The 75-gram oral glucose tolerance test (OGTT) was done for all included patients at 24-28 weeks. Women diagnosed with GDM were taken as cases and appropriately matched in equal numbers as controls. Statistical analysis was done using SPSS version 20 (IBM Corp., Armonk, NY, USA). Independent-samples t-test, chi-square test, receiver operating characteristic curve, and Pearson’s correlation coefficient (r) were performed wherever applicable.

Results

A total of 93 cases and 94 controls were included. Fetuses of women with GDM had significantly higher mean FASTT at 20 weeks (1.605 ± 0.328 mm vs. 1.222 ± 0.121 mm; p < 0.001). The FASTT cut-off obtained was 1.35 mm (sensitivity = 79.6%, specificity = 87.2%, positive predictive value = 86%, negative predictive value = 81.2%). There was a moderate positive correlation between fasting blood sugar (FBS) and two-hour OGTT values and FASTT (r = 0.332, p < 0.001 and r = 0.399, p < 0.001, respectively). FASTT >1.35 mm had an independent predictive value for GDM and was associated with a 19.608-fold increased risk of GDM.

Conclusions

FASTT values greater than 1.35 mm at 20 weeks are associated with a significantly increased risk of GDM. In addition, FASTT correlates with FBS and two-hour OGTT at 24-28 weeks and is a simple predictor of GDM at 18-20 weeks.

## Introduction

Gestational diabetes mellitus (GDM) has been defined as any degree of glucose intolerance with onset or first recognition during pregnancy [1]. GDM affects both the mother and the fetus, resulting in various adverse outcomes, particularly when it develops early [2]. It is associated with gestational hypertension, pre-eclampsia, preterm labor, increased cesarean rates, large for gestational age (LGA) fetuses, and macrosomia [3,4]. The term LGA has mainly been used for fetuses or newborns with an (estimated) weight more than the 90th percentile or more than two standard deviations from the mean for the gestational age. Macrosomia is a term mostly used for newborns with a birth weight above a certain limit. However, there is no consensus on what this limit should be. Birth weights >4,000 g, >4,200 g, and >4,500 g are used as definitions of newborn macrosomia [5-7]. In general, a fetus or newborn with an estimated or actual birth weight of more than 4,000 g appears to be macrosomic, particularly in cases of insulin-dependent diabetes mellitus (DM) [5].

With intervention, fetal complications such as macrosomia can be reduced by up to 50% [8,9]. The offspring of GDM mothers have a two to four times higher risk of future metabolic syndrome [10-12], increased body mass index (BMI), body adipose tissue composition, and subcutaneous fat [13-15]. Unfortunately, 30-40% of GDM cases remain undiagnosed until the insulin resistance peaks at 24-28 weeks of gestation, by which time the negative consequences become obvious [16].

Growth acceleration and disproportion noted in LGA fetuses of GDM pregnancies begin at around 18 weeks of gestation and are associated with an increased risk of macrosomia [17-19]. Maternal biochemical and metabolic abnormalities are not evident until 22-24 weeks, even though the fetus already exhibits signs of overgrowth, disproportion, and excessive availability of nutrients [20]. Excessive glucose transfer from a GDM mother to her fetus and placenta results in fetal pancreatic hypertrophy, hyperinsulinemia, hyperglycemia, and accelerated growth of insulin-dependent tissues, such as the liver. According to emerging evidence, increased fetal adiposity begins in early fetal life in GDM pregnancies [21,22], hence, subcutaneous fat thickness is considered a more accurate index of maternal glucose control than the biochemical glycemic profile [23].

Fetal anterior abdominal wall subcutaneous tissue thickness (FASTT) has previously been proven to be a reliable marker of fetal abdominal subcutaneous tissue adiposity [21,23,24]. We aimed to assess whether FASTT is an early sonographic sign of GDM, prior to the biochemical diagnosis of GDM at 24-28 weeks, and to study its correlation with fasting blood sugar (FBS) and 75-gram oral glucose tolerance test (two-hour OGTT) during GDM screening at 24-28 weeks.

## Materials and methods

This prospective, case-control study was conducted between July 2017 and July 2019 in the Obstetrics and Gynaecology Clinic and Fetal Medicine Department at a tertiary referral center in India. The research protocol was approved by the Institutional Ethics Committee (approval number: IRB-AIMS-2017-138).

Sample size

Based on the results of previous publications on increased fetal adiposity prior to diagnosis of GDM [25] and with 80% power and 95% confidence interval, enrolment was continued till a sample size of 93 cases and 94 controls was achieved. A total of 896 consecutive pregnancies that met the inclusion criteria were enrolled.

Selection and description of participants

All women with singleton gestations between 18 and 20 weeks without pre-gestational DM and type 1 DM were considered for enrolment. Those with multiple pregnancies, chronic kidney disease, fetal anomalies, obstetric complications (fetal growth restriction, preeclampsia, antepartum hemorrhage, intrauterine fetal demise), autoimmune disorders (antiphospholipid antibodies, systemic lupus erythematosus), hemolytic anemia, chronic hypertension, or taking medications that affect carbohydrate or lipid metabolism were excluded. At the booking visit (six to eight weeks), demographic details, maternal age, BMI, parity, gestational age, obstetric history, previous history of GDM, and a family history of diabetes were noted. Screening for pre-gestational diabetes was done in all women as per the hospital protocol and only screen-negative women were considered for enrolment.

The women underwent a fetal anomaly scan at 18-20 weeks in accordance with international guidelines at the Department of Fetal Medicine and Perinatology. The ultrasonographic fetal biometric measurements were obtained using the Hadlock formula.

Measurement technique

All measurements were obtained by an experienced fetal medicine specialist using a curved linear array transducer and two-dimensional (2D) ultrasonography on a Voluson, E10 machine (GE Healthcare, Milwaukee, WI, USA). FASTT was used as a measure of fetal adiposity and disproportion. The standard plane used to measure the AC (transverse section of the abdomen at the level of the stomach, spine, portal vein, and adrenal gland) was utilized to measure FASTT.

**Figure 1:**
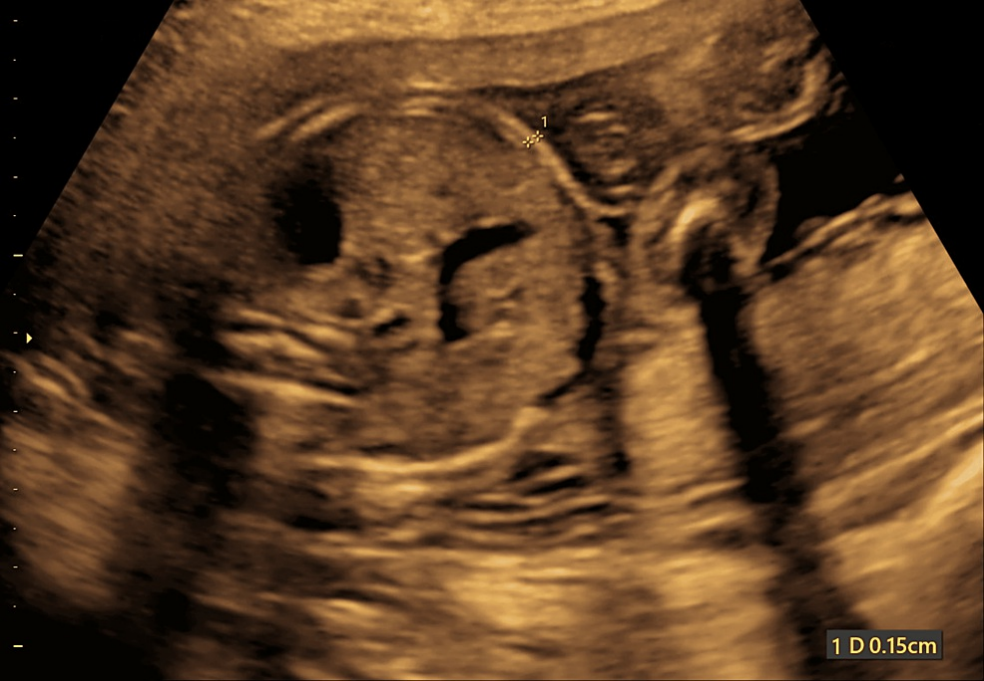
Fetal anterior abdominal wall subcutaneous tissue thickness measurement at 20 weeks. The subcutaneous tissue thickness is measured between two lines drawn tangential to its outer and inner boundaries in the anterior abdominal wall in the same section used to measure the abdominal circumference of the fetus.

FASTT was calculated as the thickness of the hyperechoic rim 3 cm lateral to the umbilical cord insertion into the portion of the anterior abdominal wall involving its one-third aspect. The distance from the outermost edge of the rim to the innermost margin of the anterior abdominal wall was measured, as shown in Figure 1. Three measurements were obtained, and the means of the measurements were calculated.

The 75-gram OGTT was performed between 24 and 28 weeks of pregnancy. GDM was diagnosed based on the Indian Association of Diabetes in Pregnancy (IADPSG) criteria, i.e., FBS ≥92 mg/dL, one-hour plasma glucose ≥180 mg/dL, or two-hour plasma glucose ≥153 mg/dL. FBS and two-hour OGTT at 24-28 weeks were recorded for each patient to study the correlation between FASTT and FBS and FASTT and two-hour OGTT.

Women who were diagnosed with GDM were taken as cases. For every case, a subsequent pregnancy from the cohort matched for age, BMI, gestational age, and parity was taken as control. Pregnancy was followed until delivery, and the data were collected. Study-specific data included BMI prior to delivery, gestational age at delivery, mode of induction, epidural requirement, mode of delivery, indication for instrumental delivery/lower-segment cesarean section (LSCS), birth weight, and the gender of the neonate.

Statistical analysis

Collected data were compiled using Microsoft Excel 2010 and analyzed using SPSS version 20.0 (IBM Corp., Armonk, NY, USA). The mean value of FASTT was calculated and analyzed between the two groups. Quantitative variables are expressed as mean ± SD and categorical variables as frequency and proportion. To test the statistical significance of FASTT between groups (GDM vs. control), an independent-sample t-test was used. To test the degree of correlation between FASTT vs. FBS and FASTT vs. two-hour OGTT, Pearson’s correlation coefficient (r) was used. Receiver operating characteristic (ROC) was used to determine the best cut-off for FASTT value to predict GDM, and the corresponding sensitivity, specificity, negative predictive value (NPV), and positive predictive value (PPV) were estimated. Pearson’s chi-square test was used to compare the categorical variables such as parity, family history of DM, and previous history of GDM within groups (GDM and control). Multivariate logistic regression analysis was done to assess independent predictors of GDM. A p-value of <0.05 was considered statistically significant.

## Results

**Figure 2:**
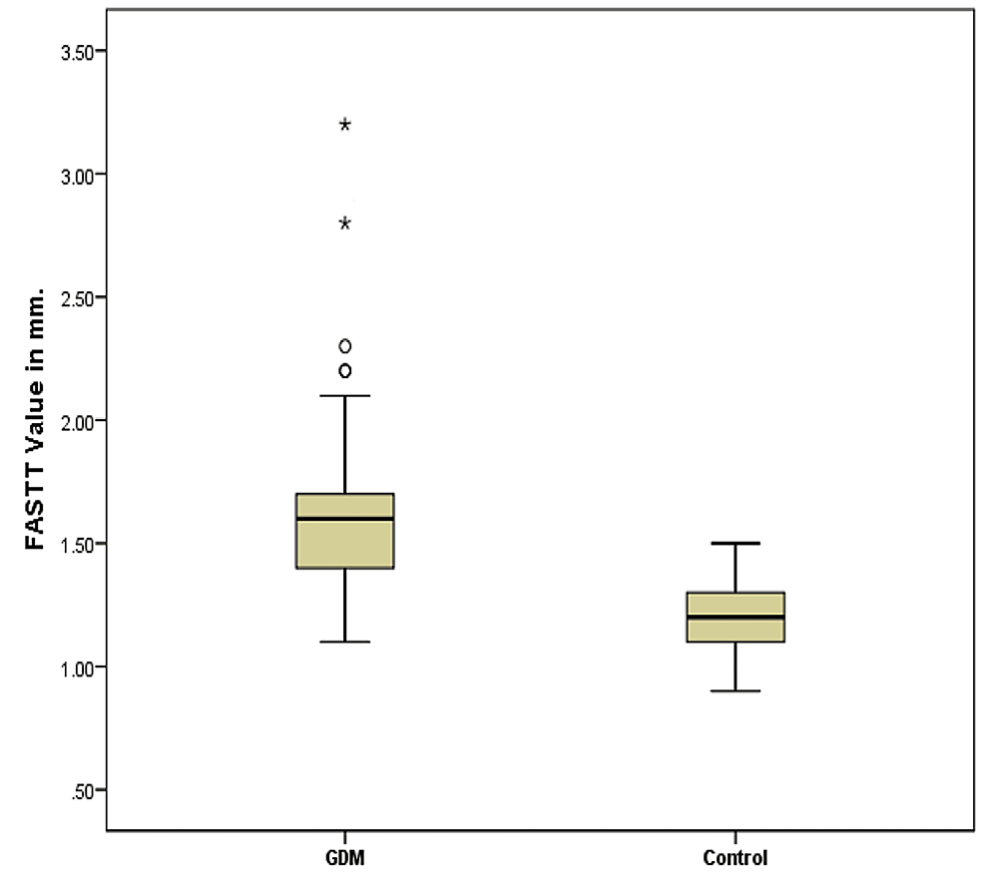
Comparison of mean FASTT value in GDM and control groups. The mean FASTT value is 1.605 ± 0.328 mm in GDM vs. 1.222 ± 0.121 mm in control (p < 0.001). FASTT = fetal anterior abdominal wall subcutaneous tissue thickness; GDM = gestational diabetes mellitus

The demographic and biometric variables of the two groups were comparable, as shown in Table 1 and Table 2. However, fetuses of women diagnosed with GDM at 24-28 weeks had a significantly higher mean FASTT value (1.605 ± 0.328 mm in GDM vs. 1.222 ± 0.121 mm in control; p < 0.001), as shown in Figure 2.

**Table 1:** Comparison of baseline characteristics in the GDM and control groups. BMI = body mass index; DM = diabetes mellitus; GDM = gestational diabetes mellitus

Characteristics	GDM group (n = 93) (Mean ± SD)	Control group (n = 94) (Mean ± SD)	P-value
Age (years)	28.87 ± 4.857	28.46 ± 4.569	0.549
Weight (kg)	70.266 ± 10.884	70.611 ± 11.066	0.830
Height (cm)	155.30 ± 5.008	155.35 ± 4.951	0.945
BMI (kg/m^2^)	29.123 ± 4.268	29.249 ± 4.352	0.842
Gestational age (weeks) at first examination	20.0031 ± 0.773	19.9156 ± 0.8515	0.464
Parity (%)			
Primigravida	34.4	34.0	0.958
Multigravida	65.6	66.0	
Mean gestational age at delivery (weeks)	37.913 ± 1.494	38.404 ± 1.354	0.040
Birth weight (kg)	3.113 ± 0.501	3.018 ± 0.459	0.179
Neonatal sex (%)			
Male	48.4	55.3	0.343
Female	51.6	44.7	
History of GDM (%)	12.9	2.1	0.012
Family history of DM (%)	36.6	22.3	0.033

**Table 2:** Comparison of fetal biometric variables between GDM and control groups at 20 weeks. AC = abdominal circumference; BPD = biparietal diameter; EFW = estimated fetal weight; FASTT = fetal anterior abdominal wall subcutaneous tissue thickness; FL = femur length; GDM = gestational diabetes mellitus; HC = head circumference

Biometric variables	GDM group (n = 93) (Mean ± SD)	Control group (n = 94) (Mean ± SD)	P-value
BPD (mm)	46.599 ± 3.336	46.383 ± 3.467	0.665
HC (mm)	170.99 ± 10.430	169.70 ± 11.179	0.417
AC (mm)	150.13 ± 11.351	147.29 ± 10.119	0.072
FL (mm)	32.74 ± 2.874	32.26 ± 2.782	0.241
EFW (g)	343.22 ± 61.257	330.74 ± 57.160	0.152
FASTT (mm)	1.605 ± 0.328	1.222 ± 0.121	<0.001

There was a moderate positive correlation between FBS and FASTT and two-hour OGTT and FASTT (r = 0.332, p < 0.001 for FBS, r = 0.399, p < 0.001 for two-hour OGTT) after adjustment for maternal age, height, weight, BMI, parity, fetal sex, and gestational age.

There was no correlation between the mean FASTT and birth weight in this study (r = 0.140, p = 0.057) because GDM patients were well-controlled on diet/oral hypoglycemic agents (OHA)/insulin. There was no correlation between mean FASTT and AC at 20 weeks in this study (r = 0.081, p = 0.270).

**Figure 3:**
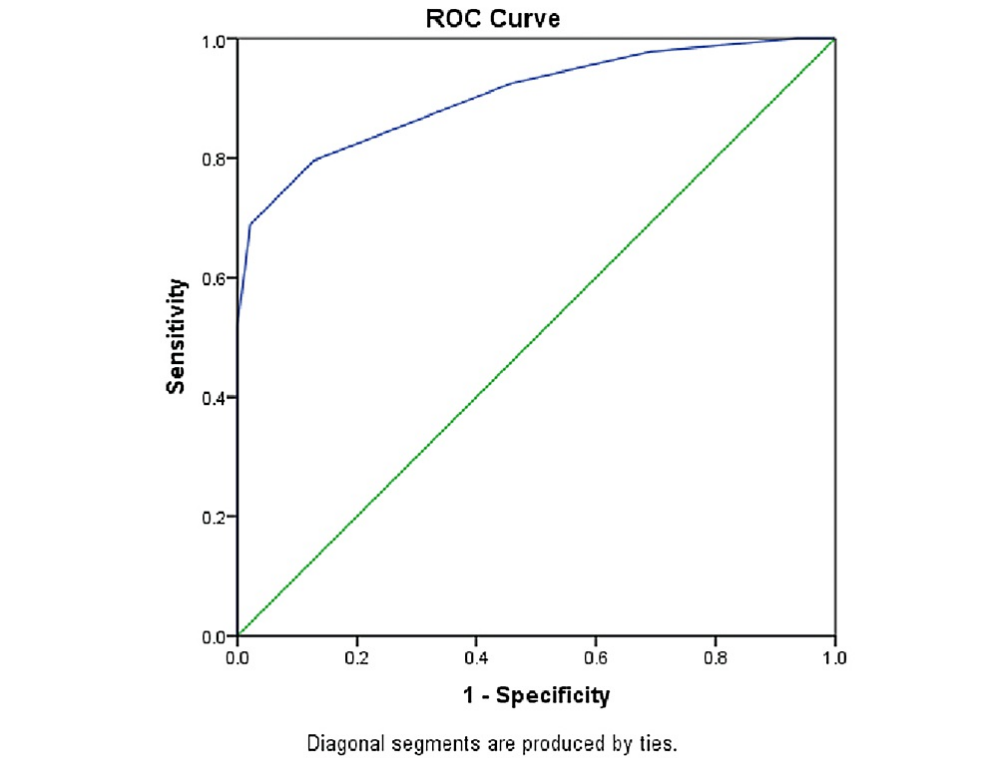
ROC curve analysis. The cut-off of FASTT for the GDM group obtained by the ROC curve was 1.35 mm. FASTT = fetal anterior abdominal wall subcutaneous tissue thickness; GDM = gestational diabetes mellitus; ROC = receiver operating characteristic

The cut-off of FASTT for the GDM group obtained using the ROC curve was 1.35 mm. The area under the curve was 0.904 (95% confidence interval = 0.861 to 0.947; p < 0.001) as shown in Figure 3. The sensitivity of FASTT >1.35 mm for predicting GDM was 79.6% and specificity was 87.2%, with a PPV of 86% and an NPV of 81.2%.

The mean gestational age at delivery in the GDM group was 37.913 ± 1.494 weeks and in the control group was 38.404 ± 1.354 weeks (p = 0.04). It showed a statistically significant difference between the two groups, as 23.7% of patients in our study were GDM on insulin who were induced and delivered earlier than the control group where we waited for the spontaneous onset of labor (as per our institutional protocol). Overall, 38.7% of patients had a normal vaginal delivery, and 61.3% underwent LSCS in the GDM group, as shown in Table 3.

**Table 3:** Frequency distribution of mode of delivery between GDM and control groups. GDM = gestational diabetes mellitus; LSCS = lower-segment cesarean section

Mode of delivery		GDM group (n = 93)		Control group (n = 94)		P-value
	Group	Number (n)	Percentage (%)	Number (n)	Percentage (%)	0.206
	Normal delivery	36	38.7	45	47.9	
	LSCS	57	61.3	49	52.1	

On multivariate logistic regression analysis, FASTT >1.35 mm had an independent predictive value for GDM and was associated with a 19.608-fold increased risk of GDM than FASTT <1.35 mm.

## Discussion

The current literature has reported higher FASTT in women with GDM in the third trimester, i.e., after the biochemical diagnosis of GDM. This study aimed to compare FASTT and fetal biometric parameters in women with GDM in the second trimester at 20 weeks. The most striking finding was that although there was no significant difference between the study groups in the standard biometric measurements, the mean FASTT value was significantly greater in the GDM group.

Growth acceleration and disproportion noted in LGA fetuses of GDM pregnancies begin at around 18 weeks of gestation and are associated with an increased risk of macrosomia [17-19]. In a retrospective cohort study of 19,377 singleton pregnancies from 16 to 20 weeks of gestation performed by Thorsell et al., larger fetuses in the second trimester were found to have an increased risk of macrosomia [17]. Moreover, Larciprete et al. found an increased mid-thigh lean mass in GDM fetuses before the definitive diagnosis of GDM, i.e., prior to the metabolic maladaptations, at 20-22 weeks [20]. According to Bernstein et al., subcutaneous fat thickness is a better predictor of maternal glucose control than the ambulatory glycemic profile [23,24].

An increased FASTT at 20 weeks of gestation was found to be associated with GDM, four to eight weeks prior to its biochemical diagnosis, when insulin resistance is maximum. In practice, patients are screened for GDM as early as the first trimester because many South Asian women who develop GDM have no risk factors at all, and detecting unrecognized pre-gestational DM is likely to be missed if we follow the standard recommendation for screening between 24 and 28 weeks of gestation [26,27]. GDM is diagnosed in 16.3% at ≤16 weeks of gestation, 22.4% between 17 and 23 weeks of gestation, and 61.3% after 23 weeks of gestation. Therefore, screening for GDM is usually done at 24-28 weeks of gestation because insulin resistance increases during the second trimester and reaches a peak at 24-28 weeks [28].

Table 4 shows a comparison of mean FASTT between GDM and control groups with previously reported studies. These results are similar to our findings, where we found a significantly increased mean FASTT in the GDM group (1.605 ± 0.328 mm) relative to the control group (1.222 ± 0.121 mm; p < 0.0001) at a gestational age of 20 weeks. The differences in the mean FASTT values, as noted in the various studies, are due to the differences in sample size and the gestational week at which the studies were conducted. Venkataraman et al. reported a significant difference between fetal biometric variables and estimated fetal weight at 20 weeks between the GDM and control group [25]. The contrasting results obtained in this study could be due to significant differences in the maternal BMI noted between the groups and a disproportionate increase in adipose tissue over lean body mass at later gestation. Catalano et al. described this differential fat distribution and a significant preferential increase in adiposity over routine biometric measurements in fetuses of GDM mothers [21]. Infant abdominal adipose tissue and liver lipid increase with increasing maternal BMI across the normal range [29]. The mean BMI in this study was comparable between both groups (29.12 ± 4.26 kg/m^2^ in GDM vs. 29.24 ± 4.35 kg/m^2^ in control), thus eliminating any bias in results based on increased adiposity reflected as increased BMI. Our results are in agreement with those reported by Gruendhammer et al. where patients and controls were matched for BMI [31].

**Table 4:** Comparison of Mean FASTT between GDM and Control groups with various studies FASTT = fetal anterior abdominal wall subcutaneous tissue thickness; GDM = gestational diabetes mellitus

Studies	Gestational age (weeks)	Sample size GDM (n), control (n)	GDM group mean ± SD (mm)	Control group mean ± SD (mm)	P-value
This study	20	93, 94	1.605 ± 0.328	1.222 ± 0.121	<0.0001
Venkataraman et al. [25]	20	153, 178	2.63 ± 0.51	2.39 ± 0.41	<0.0001
Venkataraman et al. [25]	32	153, 178	4.65 ± 0.81	4.37 ± 0.66	<0.0001
Al-Sawy et al.[32]	24–26	25, 25	6.18 ± 0.68	3.15 ± 0.53	<0.0001
Aksoy et al. [24]	26–28	55, 69	4.07 ± 0.46	3.28 ± 0.37	<0.0001
Modi et al. [29]	31	15, 16	4.4 ± 0.1	3.7 ± 0.1	<0.05
Modi et al. [29]	37	15, 16	5.6 ± 0.2	4.8 ± 0.1	<0.05
Larciprete et al. [20]	39	85, 208	6.80 ± 0.89	6.18 ± 1.32	<0.03
Tantanasis et al. [33]	24–26	20, 15	6.575 ± 0.9931	3.387 ± 0.6128	<0.0005

The cut-off of FASTT for the GDM group obtained using the ROC curve was 1.35 mm at 20 weeks. The sensitivity of FASTT >1.35 mm at 20 weeks for predicting GDM was 79.6% and specificity was 87.2%, with a PPV of 86% and an NPV of 81.2%. It is evident from the study of Al-Sawy et al. [32] and Tantanasis et al. [33] that the sensitivity and specificity of FASTT cut-off increase with gestational age. A significant moderate positive correlation between FASTT and OGTT (FBS as well as two-hour OGTT) was noted in this study as early as 20 weeks. These results correlate with the previous studies that have reported an independent relationship between maternal fasting plasma glucose level at OGTT and adiposity at birth in neonates of mothers with GDM [21,25]. In unselected pregnant populations, FASTT may become an important parameter in predicting macrosomia [34] and fetal growth restriction [35].

Petrikovsky et al. investigated the usefulness of sonographically measuring the thickness of the abdominal subcutaneous tissue in predicting fetal macrosomia in 133 term fetuses and reported that it was useful for ruling out macrosomia. A total of 113 fetuses were normal in size and 20 were macrosomic. The fetal abdominal subcutaneous tissue thickness ranged between 3 and 18 mm in all fetuses, with a mean measurement of 8.4 ± 2.7 mm (SD). The mean tissue thickness differed significantly between normal and macrosomic fetuses (7.0 mm vs. 12.4 mm, respectively; p = 0.0001) [34]. Gardeil et al. showed that measuring the abdominal subcutaneous tissue thickness is a simple and useful technique for predicting fetal growth restriction [35]. In the study by Higgins et al., a fetal anterior abdominal wall measurement greater than 3.5 mm at 30 weeks, 4.5 mm at 33 weeks, and 5.6 mm or more at 36-39 weeks of gestation or an AC >90th centile for gestation should alert the obstetrician to the possibility of fetal macrosomia [36]. In the study by Rajeshwari et al., there was a significant difference in FASTT between fetuses with normal birth weight and those who were macrosomic (7 mm vs. 12.4 mm; p = 0.0001). They obtained a FASTT cut-off value of 6.25 mm for large babies. FASTT of 6.25 mm is a sensitive test to predict LGA babies and has a high NPV [37]. Bethune et al. showed that fetal subcutaneous tissue thickness >5 mm was more useful than AC as a predictor of macrosomia in 90 pregnancies affected by GDM [38].

Several studies have examined the fetal abdominal adiposity to guide therapeutic changes (altering the dose of insulin/OHA) [18,36], as changes in fetal adiposity become evident before discernible biochemical changes. This can be an attractive prospect in terms of intervention and needs to be studied further. The mode of delivery, as evident in Table 3, and mean neonatal birth weight, as evident in Table 1, were comparable between the GDM and control groups as GDM patients were well controlled on diet/OHA/insulin. The glycemic profile was strictly monitored every week along with a growth scan every two weeks to look for any evidence of impaired glucose tolerance and accelerated fetal growth, which was immediately addressed with dose adjustment.

Aksoy et al. suggested a significant correlation between FASTT and mean birth weight [24]. Our results were in disagreement and could be the result of well-controlled GDM in our study population. It is evident that FASTT can be used to predict GDM and macrosomia, as well as to guide management in diabetic pregnancies. This is an easily reproducible, explicable, non-invasive, and cost-effective method.

Study strengths

Our study was a prospective study with strict exclusion criteria. Controls were matched with respect to age, parity, height, weight, BMI, and gestational age. Interobserver bias was eliminated as all ultrasonography measurements were done on the same machine by a single experienced fetal medicine specialist.

Study limitations

A small sample size and lack of serial FASTT measurements at various gestational weeks are the limitations of this study. More prospective studies on a large scale are needed to evaluate the efficacy of FASTT in the prediction of GDM and macrosomia, as well as as a tool to guide the management of diabetic pregnancies.

## Conclusions

We conclude that fetal adiposity evident as early as 20 weeks of gestation is associated with GDM. FASTT >1.35 mm at 20 weeks correlates with FBS and two-hour OGTT at 24-28 weeks and is a simple measurement that can be used to efficaciously predict GDM. FASTT can be used to predict macrosomia and allow intervention early in pregnancy, thereby preventing maternal and fetal complications. Increased subcutaneous tissue thickness, adiposity, and growth pattern in GDM pregnancies need to be studied in detail, particularly in larger study groups and other ethnic populations, to reinforce the importance of FASTT.
